# White Esthetic Score as a Tool for Esthetic Assessment of Tooth-Supported Restorations: A Comprehensive Review with Case Illustration

**DOI:** 10.3390/bioengineering13060690

**Published:** 2026-06-16

**Authors:** Abdulrahman Alshabib, Silvia Rojas-Rueda, Jose Villalobos-Tinoco, Khalid M. Aldosary, Francisco Garcia-Torres, Carlos A. Jurado, Mark A. Antal

**Affiliations:** 1 Department of Restorative Dental Science, College of Dentistry, King Saud University, Riyadh 11545, Saudi Arabia; 2 School of Dental Medicine, Ponce Health Sciences University, Ponce, PR 00732, USA; 3 Graduate Program in Periodontics, School of Dentistry, National University of Rosario, Rosario S2000CGK, Argentina; 4 Department of Restorative Dentistry, Centro de Estudios Odontologicos (CEO), Queretaro 76050, Mexico; 5 Restorative Department, Dental University Hospital, King Saud University, Riyadh 11545, Saudi Arabia; 6 Department of Prosthodontics and Implantology, School of Dentistry, University of La Salle, Leon 37150, Mexico; 7 Department of Operative and Esthetic Dentistry, Faculty of Dentistry, University of Szeged, 6720 Szeged, Hungary

**Keywords:** dental ceramics, esthetic score system, esthetic dentistry, veneers, white esthetic score

## Abstract

Background: The White Esthetic Score (WES) is a standardized clinician-reported index that assesses the esthetic quality of a single-tooth restoration by comparison with a natural reference tooth, typically the contralateral tooth. It evaluates five domains: tooth form, crown outline/volume, color (hue/value), surface texture, and translucency/characterization. Each domain is scored from 0 to 2 (major discrepancy, minor discrepancy, no discrepancy), yielding a total score of 0–10; higher scores indicate a closer match. Although developed for single-tooth implant restorations, WES has also been applied to natural teeth and tooth-supported restorations. Methods: This comprehensive review summarizes case-report evidence applying WES to tooth-supported restorations, outlining the concept, scoring method, documentation requirements, and available data on reliability and interpretation. A case illustration is also presented in which a patient received eight anterior veneers; outcomes were assessed using all WES parameters. Results: Published reports support WES as a practical qualitative tool to assess esthetic outcomes in tooth-supported restorations. In the presented case, the veneers achieved a WES of 9, reflecting marked improvement in tooth form, crown outline/volume, color, surface texture, and translucency/characterization. Conclusions: The comprehensive review indicates WES is feasible for routine clinical use in practice, but agreement varies by parameter and improves with standardized photography and examiner calibration; some components show lower inter-rater agreement than simpler soft-tissue indices. Because correlations between WES and patient satisfaction are inconsistent, WES should be complemented with patient-reported outcome measures. Common thresholds consider WES ≥ 6 acceptable. Clinical use for crowns and veneers should emphasize case selection, standardized records, and combined clinician- and patient-centered outcome reporting.

## 1. Introduction

Esthetic success in single-tooth restorations, particularly in the anterior maxilla, depends on more than restoration survival, marginal integrity, and biological stability [1,2]. Although these factors remain essential for long-term clinical success, the visible integration of the restoration with the surrounding dentition is often the primary factor by which patients judge the outcome. Patients typically evaluate anterior restorations during speech, smiling, and facial expression, where even minor discrepancies in shape, shade, contour, surface texture, or translucency may become noticeable. For this reason, clinicians must be able to document and communicate esthetic results in a way that is repeatable, interpretable, and comparable across patients, operators, treatment modalities, and follow-up periods [3]. Traditional descriptive evaluations, such as good, acceptable, or excellent, are inherently subjective and may vary considerably among clinicians, patients, and evaluators. These subjective descriptions do not allow meaningful benchmarking, objective comparison between studies, or reliable synthesis of clinical outcomes. Standardized esthetic indices address this limitation by converting visual impressions into structured scores that can be more consistently recorded and interpreted. In implant dentistry, the need for objective esthetic criteria became especially evident because implant success, traditionally defined by osseointegration and absence of biological complications, does not necessarily guarantee a natural-looking result. This led to the development of soft-tissue indices, such as the Pink Esthetic Score (PES), and restoration-focused indices, including the White Esthetic Score (WES), to provide a more comprehensive assessment of esthetic outcomes [4,5,6].

The WES was introduced within the PES/WES framework to objectively evaluate the “white” components of maxillary anterior single-tooth rehabilitation, specifically the visible restorative component. This scoring system was originally reported in the context of single-tooth implant replacement and early implant placement protocols, where both peri-implant soft tissue and crown morphology contribute to the final esthetic result. While the PES focuses on the peri-restorative mucosa, papillae, soft-tissue contour, and gingival architecture, WES focuses specifically on how closely the restoration resembles a natural reference tooth [7,8]. The WES evaluates restorative parameters such as tooth form, outline and volume, color, surface texture, translucency, and characterization, allowing clinicians to identify specific areas that may require improvement. Although the index was initially developed for implant-supported restorations, its restorative parameters are also relevant for tooth-supported crowns, veneers, and other anterior esthetic restorations. Lanza and colleagues later described the clinical application of the PES/WES approach to natural teeth, supporting its extension beyond implant-only contexts when an appropriate contralateral natural reference tooth is available [9]. This broader application suggests that WES may serve as a practical tool for documenting and comparing the esthetic integration of tooth-supported restorations, particularly in cases where the goal is to reproduce the appearance of the adjacent natural dentition.

Although WES is commonly reported in implant studies, clinicians increasingly seek a simple, communicable method to assess esthetics of tooth-supported single restorations such as crowns and veneers in the smile zone. Tooth-supported applications pose distinct considerations: the reference tooth may have different age-related wear, intrinsic shade gradients, or prior restorations, and the peri-restorative tissues may behave differently than around implants. A narrative review is appropriate here because WES evidence spans heterogeneous study designs (clinical trials, cohort studies, validation studies, case reports), and the goal is practice-oriented synthesis and guidance rather than pooled effect estimates. This narrative review aims to define WES and its scoring domains, summarize how WES is applied and reported, synthesize evidence on reliability, validity, and interpretive thresholds, and provide practical guidance for using WES to evaluate tooth-supported single-tooth restorations in the esthetic zone.

## 2. Materials and Methods

### 2.1. Literature Review

The White Esthetic Score (WES) is a clinical assessment system used to objectively evaluate the esthetic integration of a dental restoration with the surrounding dentition. For tooth-supported restorations, the WES focuses on the visible restorative component and compares it with the adjacent or contralateral natural teeth. The system typically assesses parameters such as tooth form, outline and volume, color, surface texture, translucency, and characterization. Each parameter is scored to provide a standardized evaluation of how closely the restoration mimics the appearance of the natural tooth structure. The present manuscript is a clinically oriented narrative review focused exclusively on evaluating the use of the White Esthetic Score in clinical scenarios involving tooth-supported restorations.

First, a literature search was conducted by two authors (A.A.; C.A.J.) to identify case reports published between January 1990 and December 2025 describing the use of the White Esthetic Score (WES) to evaluate outcomes of tooth-supported restorations. They were searched in PubMed and Google Scholar. In PubMed, the following search string was applied: ((((White Esthetic Score), (Esthetic Index), AND (WES)) OR (Pink Esthetic Score/PES)) AND (PES/WES)). This search initially identified 153 articles. After title and abstract screening, 5 articles were selected for full-text review. For Google Scholar, the search terms “White Esthetic Score,” “WES,” and “Esthetic Index” were used. As of December 2025, this search returned 46 articles. Second, following screening of titles and abstracts, 7 were retained for full-text review. Finally, only case reports providing a detailed, step-by-step description of the assessment of tooth supported restorations with the WES system were included.

Based on the inclusion and exclusion criteria summarized in Table 1, letters, books, book chapters, literature reviews, experimental studies, and articles without full-text availability were excluded. Only clinical publications including case reports and case series describing tooth fragment reattachment, with or without the use of fiber posts, were included. After removing duplicates, 12 clinical publications were included in the final analysis. It is important to mention that much of the available literature applying the White Esthetic Score focuses on implant-supported restorations. However, the present review focuses exclusively on tooth-supported restorations, because implant-supported restorations involve additional variables, such as surgical procedures, grafting, implant positioning, and soft-tissue contour. Therefore, this manuscript aims to address a gap in the literature by providing a focused review of tooth-supported restorations evaluated using the WES system.

**Figure 1:**
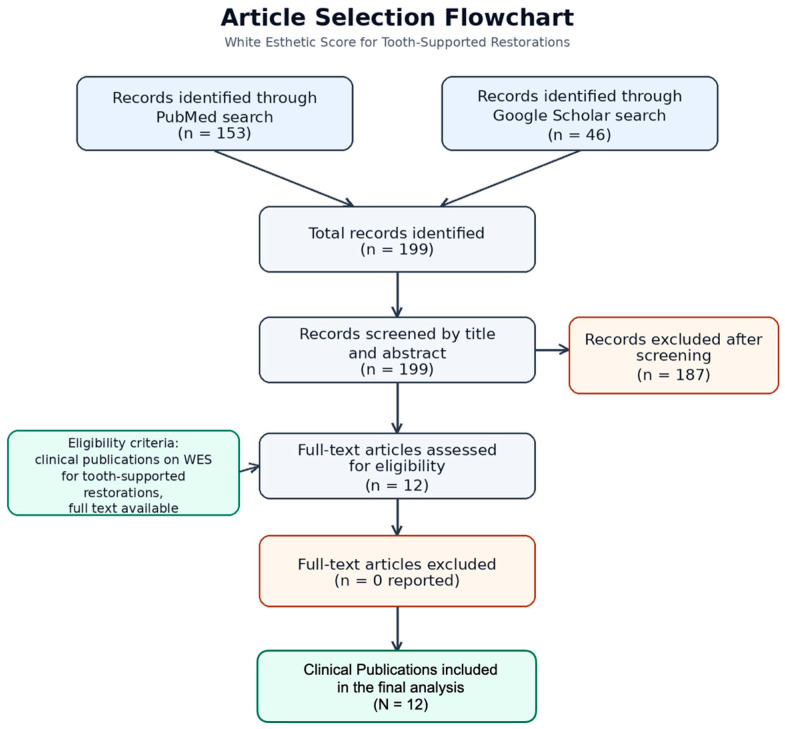
Flowchart of the article’s selection.

A flowchart of the article selection process can be seen in Figure 1.

### 2.2. Case Illustration

**Figure 2:**
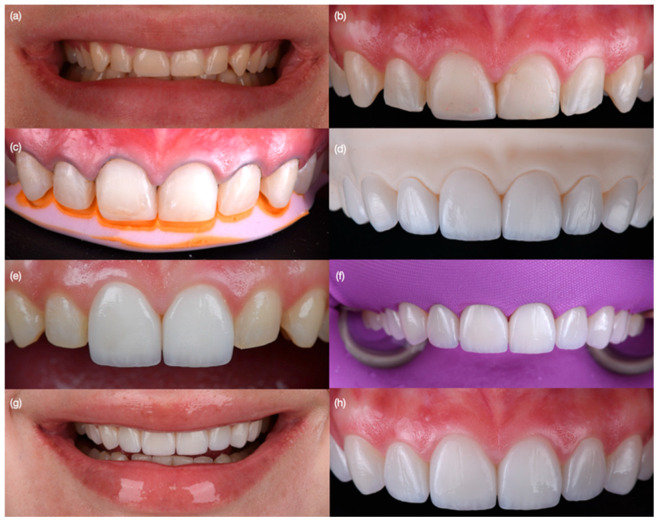
Clinical sequence of the veneer restorations. Initial (**a**) smile and (**b**) intra-oral situation, (**c**) conservative preparations with reduction guide, (**d**) fabrication of the ceramic veneers, (**e**) try-in of the veneers, (**f**) cementation of the veneers under dental dam isolation, and final (**g**) smile and (**h**) intra-oral situation.

A 35-year-old female patient presented to the dental clinic seeking improvement of her smile. She reported disliking the small spaces between her teeth and noted that several teeth appeared slightly tilted. Clinical examination revealed mild incisal wear and interdental spacing (Figure 2a,b). After clinical evaluation, the patient was diagnosed with mild incisal wear of the right canine and both lateral incisors, non-ideal incisal embrasures, and non-ideal gingival zenith positions in several anterior teeth.

The patient was offered gingivectomy to improve the zenith positions of both central incisors, tooth whitening, and minimally invasive preparations for lithium disilicate veneers from the right first premolar to the left first premolar. However, she declined both the gingivectomy and whitening procedures and requested restorative treatment only. Because of her low lip line, it was considered acceptable to proceed without gingivectomy.

An initial intraoral scan was obtained (Medit i600, Medit, Seoul, Republic of Korea), and a digital wax-up was created (3.1 Rijeka, Exocad Dental CAD, Darmstadt, Germany) to enhance tooth shape. A digital diagnostic model incorporating the wax-up was then 3D printed (Anycubic Resin 3D Printer Mono 4K, Anycubic, Shenzhen, China), and tooth reduction guides were fabricated from this model. Minimally invasive tooth preparations were completed using a specialized bur kit (Solution Laminate Veneer Preparation System, Brasseler USA, Savannah, GA, USA). A double-zero retraction cord (Ultrapak #00, Ultradent, South Jordan, UT, USA) was packed around all prepared teeth to establish equigingival finish margins, and the preparations were then polished (Sof-Lex XT Disc, 3M, St. Paul, MN, USA) (Figure 2c).

A final digital impression was taken (Medit i600, Medit, Seoul, Republic of Korea), and a 3D-printed model (Anycubic Resin 3D Printer Mono 4K, Anycubic, Shenzhen, China) was produced and duplicated to fabricate hand-crafted pressed lithium disilicate veneers (GC LiSi Press, GC, Lucerne, Switzerland). Veneer contours and margins were evaluated on the 3D-printed model (Figure 2d), and both the patient and clinician approved proceeding with fabrication. A dry try-in of the restorations was then performed (Figure 2e) to assess contours, margins, smile line, and lip support. The patient was pleased with the result of the dry try-in and approved cementation.

The restorations were first treated with hydrofluoric acid (Porcelain Etch, Ultradent, South Jordan, UT, USA) for 20 s, then rinsed and air-dried. Next, silane (Monobond Plus, Ivoclar, Schaan, Liechtenstein) was applied for 60 s, followed by adhesive (OptiBond FL, Kerr, Orange, CA, USA). After rubber dam isolation (Flexi Dam, Coltene, Altstätten, Switzerland), the teeth were sandblasted with water and 29 µm aluminum oxide particles (AquaCare, Velopex, London, UK), etched with 37% phosphoric acid (Total Etch, Ivoclar, Schaan, Liechtenstein) for 15 s, gently air-dried, and treated with primer (OptiBond FL, Kerr, Orange, CA, USA). Excess material was removed, and a light-shade luting resin cement (Variolink Esthetic LC, Ivoclar, Schaan, Liechtenstein) was applied to the ceramic veneers. Excess cement was removed, and each restoration was light-cured for 20 s from each aspect: facial, interproximal, mesial, and distal (Figure 2f). The patient was pleased with the contours, shape, and shade of the final pressed lithium disilicate veneers (Figure 2g,h). She was also provided with an occlusal guard to protect the restorations during sleep.

## 3. Results

### 3.1. Literature Review Outcomes

Across all included studies, the White Esthetic Score (WES) was consistently used as the principal index to evaluate the white esthetic score of tooth-supported restorations (Table 2) [10,11,12,13,14,15,16,17,18,19,20,21]. In anterior crowns and veneers, WES values increased significantly following treatment in prospective and randomized clinical studies, demonstrating its sensitivity in detecting improvements in tooth form, color, surface texture, and overall crown morphology. In comparative trials, WES effectively differentiated between restorative materials and digital workflows when statistically significant differences were present.

In contrast, several retrospective studies reported high and stable WES values over medium-to-long-term follow-up, supporting the ability of WES to reflect sustained white esthetic performance rather than only immediate postoperative changes. When no significant differences were detected between treatment groups, WES values remained comparable, indicating similar levels of white esthetic score across restorative approaches. The methodological characteristics of WES application are detailed in Table 3. While WES was widely implemented as an esthetic outcome measure, the reporting of examiner calibration and blinded scoring was inconsistent. Nevertheless, when reliability testing was performed, WES demonstrated reproducibility and examiner agreement, reinforcing its role as a structured and clinically applicable white-esthetic evaluation tool for tooth-supported restorations.

A potential risk of publication bias should also be considered. Since this review included published clinical reports and studies evaluating the White Esthetic Score in tooth-supported restorations, cases with favorable esthetic outcomes may be more likely to be submitted and published than cases with poor or unsuccessful esthetic results. This may lead to an overrepresentation of positive WES outcomes and limit the ability to fully estimate the variability of esthetic performance in routine clinical practice. Therefore, future studies should include larger prospective clinical investigations with standardized reporting of both successful and less favorable outcomes.

Moreover, the present article is a comprehensive narrative review focused specifically on clinical studies, including case reports, case illustrations, retrospective clinical studies, and randomized clinical trials that used the WES system to evaluate tooth-supported restorations in real patients. Although this review is not a systematic review, narrative reviews are an accepted and valuable type of scholarly review in the literature, particularly when the goal is to synthesize clinically relevant information, summarize heterogeneous study designs, and translate complex clinical findings into an accessible format. Future reviews should also consider including in vitro studies that evaluate the application of the WES system in laboratory settings. In addition, systematic reviews could be useful in the future because they may reduce bias, provide a higher level of evidence, and allow more rigorous quantitative analysis when sufficient homogeneous data are available. However, the present comprehensive narrative review provides value by contextualizing clinical findings with expert interpretation and offering practical information in areas where quantitative data or rigid systematic evidence may be limited.

### 3.2. Outcome of Case Illustration

A thoughtful clinical assessment is essential whenever dental care is needed in the smile zone because small imperfections can be easily noted by the patient. The presented case illustration started with diagnostic records such as diagnostic digital impressions, periodontal examination and intra-oral photos. The initial records allow the clinician to evaluate the initial outcome and discuss the areas that need to be addressed with the patient. Digital tools such as intra-oral scan and digital wax-up also help to communicate to the patient the initial situation and the proposal of the treatment. Minimally invasive tooth preparations were performed with the use of tooth reduction guides. Then polishing of the final preparations ensures smooth surfaces. A final digital impression was obtained to capture the preparations. In order to obtain highly esthetic results, hand-crafted restorations were fabricated with the pressing technique and with lithium disilicate ceramic.

**Figure 3:**
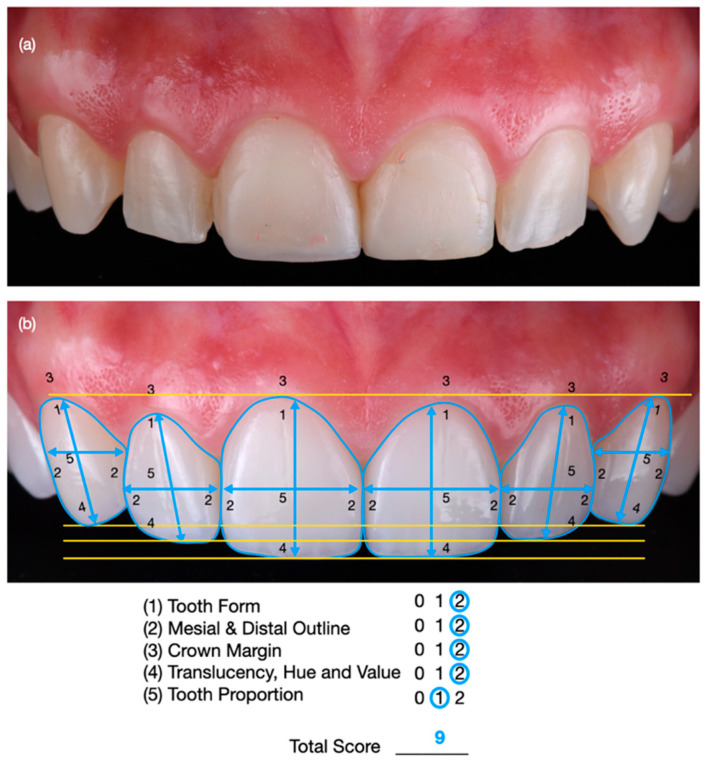
White Esthetic Score (WES) system used to evaluate the outcome of the restorations. (**a**) Initial and (**b**) final outcome with the WES evaluation with a score of 9 points.

The White Esthetic Score (WES) system was used to evaluate the outcome, and as previously mentioned, this assesses five parameters: tooth form; mesial/distal outline; crown margin; translucency in the incisal third and hue/value in the middle third; and tooth proportions. The outcome obtained in this case scenario led to a WES of 9, which represents an excellent outcome; the only parameter that did not obtain the highest score was the tooth proportions, because patient initially came with the zenith position of the left central incisor lower than the right central incisor, and because patient did not allow gingival recontouring, compromising an excellent result; however, a score of 9 is highly esthetic and due to the lip line hiding the zenith position, the small differences in tooth proportions cannot be seen in a regular smile (Figure 3).

## 4. Discussion

The White Esthetic Score (WES) translates complex esthetic judgments into a structured, reproducible, and teachable framework by directing clinicians and dental technicians to the five domains most likely to determine the detectability of a single restoration: tooth form, contour, color, surface texture, and translucency or characterization [22]. These parameters are essential in anterior esthetic dentistry because even minor discrepancies between the restoration and the adjacent natural dentition may be perceived by the patient during smiling, speech, or facial expression. By organizing the esthetic evaluation into individual categories, WES allows clinicians to move beyond general subjective descriptions, such as “good” or “acceptable,” and instead identify the specific restorative features that contribute to the final esthetic outcome. This makes the index useful not only for clinical documentation, but also for communication among clinicians, dental technicians, students, and researchers.

One of the main strengths of WES is its practicality, since it does not require specialized equipment and can be applied chairside, from standardized clinical photographs, or as part of quality assurance protocols in teaching clinics. This simplicity has contributed to its widespread use in clinical reporting and makes it especially valuable in daily practice, where clinicians need efficient tools to evaluate esthetic outcomes. In addition, WES is highly actionable because low scores can be linked to specific corrective strategies. Problems related to tooth form or outline may indicate the need for diagnostic wax-up modification, digital design correction, contour refinement, or improved laboratory communication. Color discrepancies may require more detailed shade mapping, better control of ceramic thickness, appropriate cement selection, or replacement of the restoration when necessary. Similarly, deficiencies in surface texture, translucency, or characterization may be addressed through refinishing, adjustment of glaze or polishing protocols, or modification of internal and external characterization. Therefore, WES not only provides a numerical esthetic score, but also serves as a practical guide for identifying and improving the specific factors that influence the integration of tooth-supported restorations.

WES does not evaluate soft tissue. For single-tooth restorations in the anterior zone, esthetics depend on both the crown and the surrounding tissues. WES alone can be high while the overall esthetic result is compromised by papilla loss or mucosal recession; this is why PES and combined PES/WES are commonly reported together [23,24].

The score is still observer-dependent. While WES is “objective” in the sense of structured scoring, it remains a perceptual rating scale. Reliability varies across parameters and across observers, particularly for optical domains [25].

Reference-tooth dependence can be a weakness. Contralateral teeth may be naturally asymmetric, worn, or previously restored. In tooth-supported restorative cases, this is common (e.g., unilateral fracture with contralateral incisal wear). Without careful reporting, WES can penalize a restoration that is actually esthetically improved relative to baseline.

WES is not a full color science tool. It compresses color into a clinical judgment (hue/value), but does not quantify chroma, metamerism, or fluorescence. Thus, WES should be supplemented by standardized photography and, when relevant, instrumental shade assessment (spectrophotometry) in high-demand cases [26].

### Recommendations for Applying WES to Tooth-Supported Restorations

Below is a clinically oriented workflow that improves consistency and usefulness of WES in tooth-supported single restorations:

*Documentation standardization*

Standardized documentation is essential for achieving a reliable WES assessment. Pre- and post-treatment photographs should be obtained with the contralateral tooth visible so that an appropriate reference can be used during evaluation. Whenever possible, camera settings and lighting conditions should be kept consistent, or a standardized intraoral photography protocol should be followed [27]. Cross-polarized photographs may also be considered for shade mapping, as they can provide additional information regarding color characterization. When translucency and surface characterization are especially important, a standardized close-up image of the incisal region may be helpful to improve assessment accuracy.

*Examiner strategy*

The number and calibration of examiners should depend on the clinical or academic setting. In routine private practice, scoring by a single calibrated clinician may be sufficient for internal quality assurance. However, in teaching programs and scientific publications, the use of two or more evaluators is preferable. When possible, inter-rater agreement should also be reported, given the known variability that can occur across indices, variables, and evaluators [28]. This approach strengthens the reliability of the esthetic assessment and improves the scientific value of the reported outcomes.

*Reporting strategy*

When WES is used in clinical documentation or scientific publication, the reporting should be detailed enough to allow meaningful interpretation. The total WES, ranging from 0 to 10, should be provided along with the individual scores for each of the five domains. It is also important to specify which reference tooth was used and whether that tooth was unrestored [29]. The time point of evaluation should be clearly reported, such as immediate postoperative assessment, 6 months, or 1 year. If PES/WES is used as a combined evaluation system, both the Pink Esthetic Score (PES) and WES should be reported separately. In addition, the acceptability threshold used in the study or clinical report should be clearly stated, for example, WES of 6 or greater, or a combined PES/WES of 12 or greater.

*Pairing WES with Patient-Reported Outcomes*

Because the correlation between clinician-based esthetic indices and patient satisfaction is not always consistent, WES should ideally be combined with a patient-reported outcome measure, such as a visual analog scale for overall esthetic satisfaction [30]. This is particularly important in tooth-supported esthetic dentistry, where patient expectations, self-image, and preoperative psychosocial factors may strongly influence the perception of success. Combining clinician-based scoring with patient-reported outcomes provides a more comprehensive evaluation of treatment results.

*Interpreting “Acceptable” and “Excellent” in Clinical Terms*

For tooth-supported restorations, the most useful interpretation of WES is not based on a single cutoff value, but rather on a tiered clinical approach. A WES of 9 to 10 may be interpreted as near-perfect integration, with only minimal or barely perceptible discrepancies. Scores between 6 and 8 are generally considered clinically acceptable, although some identifiable differences may still be present, often involving one or two parameters. A WES of 5 or below suggests that the esthetic mismatch is likely to be noticeable and that corrective action may need to be considered [31,32]. This approach is consistent with how WES has been subclassified in clinical reporting as poor, acceptable, or almost perfect, and it also aligns with the frequent use of 6 as the lower threshold for acceptability.

When PES is recorded together with WES, combined thresholds may be used to describe the overall acceptability of both hard and soft tissues. For example, a combined PES/WES of 12 or greater may indicate overall clinical acceptability. More recent work has also suggested additional perceptual thresholds, with values around 17 being associated with exceptional esthetic outcomes.

*Future Directions and Research Needs, Especially for Tooth-Supported WES*

Future research is needed to strengthen the application of the White Esthetic Score (WES) for tooth-supported restorations. First, formal validation of WES in tooth-supported clinical scenarios remains necessary. Although the use of WES on natural teeth and tooth-supported restorations has been described and appears clinically feasible, larger prospective studies are needed to evaluate its reliability across different restorative materials, treatment modalities, and clinical conditions.

Second, future investigations should focus on improving the reliability of individual WES parameters. Identifying which domains, such as color, translucency, surface texture, or tooth form, are most prone to disagreement among evaluators would help improve examiner calibration and scoring consistency. Standardized photographic protocols, including consistent lighting, angulation, magnification, and possibly cross-polarized photography, may also improve the reproducibility of WES assessments.

Another important area for future research is the determination of the minimal clinically important difference for WES. Clinicians need to understand whether a 1-point or 2-point change in WES represents a clinically perceptible or meaningful esthetic improvement. Recent studies evaluating perceptual thresholds in combined PES/WES scoring represent an important step in this direction, but additional research focused specifically on tooth-supported restorations is still required.

Finally, integration with digital dentistry and artificial intelligence may further enhance the clinical usefulness of WES. Automated or semi-automated analysis of contour, tooth proportions, texture, shade, and translucency could reduce observer variability and improve efficiency. These tools may be especially valuable in teaching clinics, digital smile design workflows, multicenter clinical studies, and long-term follow-up evaluations.

*Limitations of the White Esthetic Score System*

Although the White Esthetic Score is a useful and structured tool for evaluating the esthetic integration of tooth-supported restorations, it has several limitations. The score remains observer-dependent and may vary according to examiner experience, calibration, photographic quality, and interpretation of optical parameters such as color, translucency, and surface texture [33,34]. In addition, WES does not evaluate soft-tissue esthetics, patient satisfaction, or functional outcomes, and its accuracy depends on the availability of an appropriate reference tooth, which may be asymmetric, worn, discolored, or previously restored. Therefore, WES should be used as part of a broader esthetic assessment that includes standardized photography, clinical judgment, and patient-reported outcomes.

## 5. Conclusions

The White Esthetic Score (WES) is a structured and clinically useful index for evaluating the esthetic outcome of single-tooth restorations by comparison with a natural reference tooth. Although it was originally developed for anterior implant restorations, it may also be applied to tooth-supported restorations when an appropriate reference tooth is available. A score of 6 or higher is generally considered clinically acceptable. The case presented achieved a WES of 9 out of 10, indicating a highly esthetic outcome that was consistent with the patient’s complete satisfaction with the esthetic result of the veneer restorations.

## Figures and Tables

**Table 1:** Inclusion and exclusion criteria of the search performed.

Criterion	Inclusion	Exclusion
Time period	Publications available between January 1990 and December 2025	All publications published before January 1990
Language	English	Non-English
Type of articles	Clinical publications only (case reports, case series, retrospective clinical studies and randomized clinical studies) describing tooth fragment reattachment. Full text available.	Letters, books, book chapters, literature reviews, experimental in vitro studies, and articles without full text availability.

**Table 2:** Summarizes the study design, restoration type, sample characteristics, documentation methods, and main WES-related findings of the included investigations [10,11,12,13,14,15,16,17,18,19,20,21].

First Author, Year	Study Design	Restoration Type	Sample	Documentation	Main Esthetic Findings
Lanza A, et al., 2017 [10]	Case report + literature review	Tooth-supported single crown in anterior maxilla	1 patient, 1 natural tooth crown, 5-year follow-up	Standardized photographs, comparison with contralateral tooth	White Esthetic Score (WES) for the anterior single-tooth crown remained within an acceptable range over 5 years, with only slight changes in White Esthetic Score, while soft-tissue scores stayed stable; the report supports PES/WES as a reliable system for natural-tooth crowns.
Sierra D, et al., 2022 [11]	Retrospective clinical study, 6-year follow-up	Tooth-supported minimally invasive full-mouth rehabilitations (direct composite resins, indirect composite resin/ceramic onlays, and resin/ceramic veneers)	19 patients, 406 restorations (149 direct composite resins, 110 indirect composite/onlays, 147 resin/ceramic veneers), mean follow-up 71.8 ± 28.6 months	Clinical periodontal parameters (PPD, PI, BOP), tooth vitality and sensitivity; esthetic outcomes rated with the White Esthetic Score (WES); PROMs assessed using visual analog scales	Mean WES at long-term follow-up was 8.4 ± 1.9, indicating high white-esthetic quality of the minimally invasive full-mouth restorations; no secondary caries or loss of abutment tooth vitality were detected.
Merchant A, et al., 2022 [12]	Prospective clinical study	Tooth-supported anterior crowns	Patients receiving anterior crowns (exact N in article)	Pre- and post-crown clinical/photographic evaluation	Mean WES increased from 5.40 to 7.72 after placement of anterior crowns, demonstrating improvement in WES; the total esthetic score rose from 13.15 to 15.70, whereas the PES change was clinically but not statistically significant.
Deng XL, et al., 2023 [13]	Retrospective clinical study	Tooth-supported porcelain veneers in anterior region	64 patients, 152 anterior teeth	Pre- and post-treatment photographic assessment by calibrated examiners	Mean WES significantly increased from 6.85 ± 1.87 to 9.81 ± 0.58 after veneer placement, confirming marked enhancement of WES; PES also rose from 9.63 ± 2.23 to 13.64 ± 0.88 (*p* < 0.01).
Dash S, et al., 2024 [14]	Randomized clinical trial	Tooth-supported single crowns (PFM, monolithic zirconia, lithium disilicate) in anterior maxilla	45 patients, 45 crowns (3 material groups)	Standardized photographs scored twice by 8 observers from 4 specialties	WES contributed to higher overall PES/WESs in zirconia and lithium disilicate crowns compared with PFM, with zirconia achieving the highest mean combined score (~16.7); intra- and inter-observer agreement for PES/WES scoring was high.
Dong K, et al., 2024 [15]	Retrospective clinical comparative study	Tooth-supported anterior all-ceramic crowns with glass fiber post-core (experimental) vs. cobalt alloy post-core porcelain crowns (control)	121 anterior teeth: 61 glass fiber post-core all-ceramic crowns, 60 cobalt alloy post-core porcelain crowns, 12-month follow-up.	Clinical repair outcomes, esthetic effect evaluated by Pink Esthetic Score (PES) and White Esthetic Score (WES)	WES values did not differ significantly between glass fiber post-core all-ceramic crowns and cobalt alloy post-core porcelain crowns before or after treatment, indicating comparable white-esthetic outcomes for both post-core systems, despite better biologic performance in the glass fiber group.
Ren L-J, et al., 2024 [16]	Randomized controlled clinical trial	Tooth-supported anterior temporary crowns combined with esthetic crown lengthening (3D-printed double positioning guide vs. traditional restoration approach)	20 patients, 70 anterior teeth (36 in 3D-printing group, 34 in traditional restoration group), follow-up at 1, 3, and 6 months	Clinical evaluation using papilla index score (PIS), Pink Esthetic Score (PES), White Esthetic Score (WES), and patient satisfaction questionnaires at baseline and follow-up visits	WESs at 1, 3, and 6 months were significantly higher in the 3D-printing guide group than in the traditional restoration group (*p* < 0.05), showing superior WES for anterior temporary crowns fabricated with the 3D-printed double positioning guide.
Shenoy A, et al., 2024 [17]	Double-blind randomized crossover clinical trial	Tooth-supported CAD-CAM provisional restorations for full-mouth rehabilitation (same design, fabricated using CBCT vs. intraoral scanner workflows)	12 participants requiring full-mouth rehabilitation, each receiving two sets of provisional restorations (CBCT and IOS) with a washout period	Esthetic outcomes were rated by clinicians using PES, WES, and modified USPHS criteria, and by patients with OHIP-Aes and Orofacial Esthetic Index questionnaires.	Clinician-reported WES values were significantly higher for CBCT-fabricated CAD-CAM provisionals than for IOS-fabricated ones (mean rank WES: 17.25 vs. 7.75; *p* < 0.001), indicating better WES with the CBCT workflow, while patient-reported esthetic scores were similar between methods.
Srivastava G, et al., 2025 [18]	Validation study	Single anterior maxillary tooth-supported prostheses made of 3 materials	Sample of tooth-supported crowns (N reported in article)	Photographic scoring by multiple observers	WES showed good intra- and inter-observer reliability, whereas indices such as ICAI produced lower esthetic scores and slightly less consistent results, supporting WES as a reproducible white-esthetic index.
Cai F, et al., 2025 [19]	Randomized controlled trial	Tooth-supported posterior single crowns (NPJ-printed zirconia vs. CAD/CAM-milled zirconia)	43 participants, 48 posterior teeth, 24 crowns per group, 12-month follow-up	Clinical examination using FDI criteria, Pink Esthetic Score (PES), White Esthetic Score (WES), periodontal parameters, and patient satisfaction questionnaires	WES values were comparable between NPJ-printed and CAD/CAM-milled zirconia posterior crowns throughout the 12-month follow-up, with both groups maintaining high white-esthetic scores, while only minor differences appeared in pink-esthetic and clinical parameters.
Bäumer AM, et al., 2025 [20]	Retrospective clinical study	Tooth-supported anterior ceramic veneers	68 patients, 312 veneers, mean follow-up ≈ 8 years	Clinical evaluation using Pink Esthetic Score (PES) and White Esthetic Score (WES), plus patient-reported outcomes (OHIP-G14 and PIDAQ questionnaires)	WES values for anterior ceramic veneers remained high and did not differ significantly across periodontal status groups, indicating stable WES of veneers over an average of 8 years, in line with high patient satisfaction and oral health-related quality of life.
Xu Y, et al., 2025 [21]	Randomized clinical trial	Tooth-supported anterior crowns with minimally invasive laser crown lengthening (control: laser alone; experimental: laser + digital smile design)	108 patients receiving crown restoration treatment, 54 in control group and 54 in experimental group, evaluated up to 1 year	Periodontal and restorative parameters (PLI, PD, BI, RM–BC, RM–GM, tooth mobility), Liébart smile line classification, and esthetic assessment using Pink Esthetic Score (PES) and White Esthetic Score (WES)	At 6 months, WESs (and PESs) in the experimental group receiving digital smile design plus laser crown lengthening were significantly higher than in the laser-only control group (*p* < 0.05), demonstrating improved WES of anterior crowns without increasing adverse events at 1-year follow-up.

**Table 3:** Methodological Characteristics and Application of the White Esthetic Score (WES) in Tooth-Supported Restorations. Having + (plus) as low and – (minus) as high-risk bias [10,11,12,13,14,15,16,17,18,19,20,21].

Study	WES PRIMARY Outcome	Separate WES Reporting	Calibrated Examiners	Blinded WES Assessment	Pre/Post WES	Long-Term WES Follow-Up	Overall, WES Assessment
Lanza A, et al., 2017 [10].	−	+	−	−	−	+	+
Sierra D, et al., 2022 [11].	+	+	−	−	−	+	+
Merchant A, et al., 2022 [12]	+	+	−	−	+	−	+
Deng XL, et al., 2023 [13].	+	+	+	−	+	−	+
Dash S, et al., 2024 [14].	+	+	+	+	−	−	+
Dong K, et al., 2024 [15].	−	+	−	−	−	+	+
Ren L-J, et al., 2024 [16].	−	+	−	−	+	−	+
Shenoy A, et al., 2024 [17].	+	+	−	+	−	−	+
Srivastava G, et al., 2025 [18].	−	+	+	+	−	−	+
Cai F, et al., 2025 [19].	−	+	+	+	−	+	+
Bäumer AM, et al., 2025 [20].	−	+	−	−	−	+	+
Xu Y, et al., 2025 [21].	−	+	−	−	−	+	+

## Data Availability

Data presented in this study are available on request from the corresponding authors.
